# Student and Faculty Satisfaction with Their Dental Curriculum in a Dental College in Saudi Arabia

**DOI:** 10.1155/2020/6839717

**Published:** 2020-04-08

**Authors:** Maha Abdelsalam, Tobias E. Rodriguez, Lynn Brallier

**Affiliations:** ^1^Department of Biomedical Science, College of Dentistry, Imam Abdulrahman Bin Faisal University, Dammam, Saudi Arabia; ^2^The Academy for Advancing Leadership (AAL), Atlanta, USA; ^3^Gladys L. Benerd School of Education, The University of the Pacific, 3601 Pacific Avenue, Stockton, California, USA

## Abstract

The existing dental curriculum is taught at the College of Dentistry since 2002. The aim of this research is to explore the satisfaction levels of faculty members and students with that curriculum. This information will justify a curriculum reformation plan that addresses the aspirations of both faculty members and students. In this cross-sectional study, a two-section survey was prepared. Section 1 investigated the level of satisfaction with the curriculum, and Section 2 sought reasons why participants were satisfied with the curriculum. The questionnaire was electronically mailed to faculty members, interns, and senior students. Data were analyzed to identify patterns and points of disagreement expressed by faculty and students. The overall response rate was 68.7%. The mean standard deviation (SD) score in the study sample from all respondents was 5.0 (+3.0). Faculty significantly registered higher satisfaction than students (mean (SD) = 5.9 (+2.9) and 4.1 (+2.8), *P* = 0.002). Results of multivariate analysis showed that faculty members were more satisfied with the curriculum than students because they thought the curriculum prepared competent graduates (regression coefficient = 1.76 and 0.69). Teaching staff and students' satisfaction levels with the curriculum were significantly associated with their perception that the curriculum produces competent graduates. Areas with low students' satisfaction levels were related to promotion of engagement with others and development of critical thinking and problem-solving skills. These areas should be the focus of future curriculum reformation to prepare competitive graduates with competences aligned with the recommendations of the Saudi Arabia Qualification Framework and of the international benchmarks.

## 1. Introduction

Curriculum development must consider several factors including graduate qualifications, guidelines of the national accrediting body, and international standards of best practices. Faculty and students represent internal stakeholders whose satisfaction and feedback should always be sought for monitoring the quality of curricula and for planning modifications and reforms.

Although there have been colleges of dentistry in Saudi Arabia (SA) since the 1970s, a national guideline for dental programs was created only as recently as 2009 [1]. The National Center for Academic Accreditation and Evaluation in SA was established in response to the increased number of public and private colleges throughout the Kingdom and from the demand by the Ministry of Education, that institutions and programs be accredited. In SA, many dental colleges developed their curricula according to agreements with international institutes [2]. Recently inducted programs seek curricula that correspond to contemporary advances in dental education [3]. The College of Dentistry (COD) at Imam Abdulrahman Bin Faisal University (IAU) was started in 2002, applying a program that was developed based on guidance from other similar programs in the Kingdom at that time. The six-year curriculum includes discipline-specific courses and comprehensive care practice components, followed by one year of clinical rotations, referred to as internship. The curriculum has been delivered as semester-based, credit-hours program since it began.

The COD curriculum is teacher-focused with minimal elements of problem-based and case-based learning (PBL and CBL) where students get the opportunity to interact with the teacher and with each other while they learn. Dental institutes must carefully design their curricula to address the anticipated graduate attributes with consideration of the available resources. However, the question arises as to whether graduates of different curriculum types' exhibit different attitudes and practices in their professional life. In a traditional curriculum, the students are inactive participants in lectures and lectures can be seen as repetitious and focused on memorization, which is unmotivating for students. In active curriculum types, such as in the PBL and CBL models, the teachers encourage small group tutorials with students and faculty, as well as reciprocal student-faculty evaluation. The multidisciplinary experienced teaching staff and the resources required to implement such methods may pose challenges, especially when students' numbers are high [4]. Another approach employing the dialogue style in teaching microbiology course to dental students was assumed to receive high satisfaction. On the contrary, the study showed that students preferred listening to teachers who know the material. This led to switching to more traditional teaching with maintenance of the dialogue principle to a certain degree [5].

If a program targets building the drive for continuous learning and improvement in its students, then teaching methods must ensure students' interaction to achieve the desired participation and develop their commitment to acquisition of knowledge and skills through lifelong learning [6]. This was confirmed in a study of the US National Dental Boards examinations, which revealed that students in a hybrid curriculum performed significantly superior to their peers working in a traditional lecture-based curriculum [7]. In Dublin, however, there was insignificant difference between graduates of hybrid curriculum and other types of curricula to participate in continuing education courses [8].

At Imam Abdulrahman Bin Faisal University (IAU), through the annual students' learning environment and program evaluation surveys, students are able to express their satisfaction level with their education. As students progressed in the program and began to graduate, program evaluation surveys consistently showed several challenges. Those difficulties have been dealt with individually by course directors, department chairmen, or the vice dean for academic affairs. This led to the need for a school-wide, overall assessment of the satisfaction levels of the teaching faculty and students with the curriculum. This research seeks to identify the strengths and weaknesses of the curriculum so that corrective actions can be instituted through modifications in specific areas or through major curriculum reformation. We chose to analyze the curriculum through the responses of teaching staff and students to the same questionnaire, so that we could obtain a balanced perspective from both stakeholder groups. The aim of the present study is to assess the satisfaction level of teaching staff and senior students/interns with various aspects of the curriculum and to investigate their perception of the reasons behind their level of satisfaction.

## 2. Materials and Methods

### 2.1. Design and Setting

A questionnaire was used to collect data about curriculum satisfaction. Teaching staff, senior students, and interns were surveyed.

The study was conducted in the COD, IAU, SA during the academic year 2015-2016.

### 2.2. Target Groups

In Saudi Arabia, dental programs consist of six years followed by one year of clinical rotations internship. The study included all 66 teaching staff present in the College during the study period and all 78 senior dental students (5th and 6th year students and interns). All subjects of the target groups received the survey and were invited to participate in the study.

### 2.3. Survey

A survey was developed in English for the teaching staff (many of whom were not native Arabic speakers) and in Arabic for the students and interns. The survey was divided into two sections.  Section 1 in the survey assessed the satisfaction with various aspects of the curriculum using 12 statements that participants were asked to respond to on a 5-point Likert scale ranging from 1 (strongly dissatisfied) to 5 (strongly satisfied).  Section 2 in the survey investigated participants' perception of why the curriculum was satisfactory using ten statements. The respondents were asked to agree with on a scale ranging from 1 (strongly disagree) to 10 (strongly agree).

The face and content validity of the items assessing satisfaction with curriculum were checked by two teaching staff members not involved in the study. They were found to have very good internal consistency (Cronbach's alpha = 0.82).

The survey was uploaded to SurveyMonkey and emailed to all faculty members, senior students, and interns. There was no obligation, and total anonymity of participants was kept. The study was approved by the Institutional Review Boards of both IAU and University of the Pacific (IRB Proposal #15–105). With approval of the University of the Pacific IRB as “exempt,” the survey was administered to the faculty members, predoctoral senior dental students, and interns at COD. After a week, a second reminder was sent with the survey to the same e-mail accounts. The survey link was kept open for two weeks before being closed.

### 2.4. Data Analysis

Responses “strongly satisfied” and “satisfied” were recorded to indicate satisfaction. The number and percent of teaching staff and students who were satisfied were calculated, and the odds ratio of satisfaction was calculated for teaching staff compared to students. A total satisfaction score was developed by counting the number of responses indicating satisfaction among the 12 statements from each respondent. Thus, the total satisfaction scores potentially ranged from 0 to 12. A two-tailed *T*-test was used to compare teachers and students regarding total satisfaction score. Another *T*-test was also used to compare individual scores given to the potential reasons the curriculum is adopted.

Regression analysis was used to assess the effect on the satisfaction score (dependent variable) of various reasons respondents think the curriculum is satisfactory as well as whether the respondent was a teacher or a student. Significance level was set at 5%. Statistical analysis was performed using SPSS version 17.0.

## 3. Results

Of 144 that received the survey, a total of 99 responded (68.7%). Among those, 48 were teaching staff and 51 were students (72.7% and 61.4% response rates, respectively).

The mean (SD) satisfaction level score of all respondents was 5.0 (3.0), min−max = 0−12. Overall, respondents attributed their satisfaction with the curriculum to the fact that it allowed continuity of training, developed communication skills, developed critical thinking, and had integrated courses (odds ratio for satisfaction with the 4 aspects of the curriculum = 3.56, 2.80, 2.60, and 2.32).

Table 1 shows how students-and-faculty satisfaction levels with the curriculum were associated with being a teaching staff/student and with their ratings of the reasons it is satisfactory. In univariate regression, there was a significant association between satisfaction and respondents' perceptions of six out of the ten reasons the curriculum was initially developed. They were more satisfied if they thought the curriculum was being developed because it (1) fits the training of the teaching staff; (2) prepared competent graduates; (3) fulfilled the mission and vision of the college; (4) reflected college tradition; (5) followed best practices in dental education; or (6) was based on negotiations between departments (regression coefficients = 0.35, 0.71, 0.40, 0.50, 0.44, and 0.32).

In multivariate regression data analysis, the only reason the curriculum is satisfactory was because it prepared competent graduates. This factor increased satisfaction levels by 0.69 points (regression coefficient = 0.69). Teaching staff satisfaction level was about 2 points more than students' (regression coefficient = 1.74).

Teaching staff had a significantly higher satisfaction level score than students (mean (SD) = 5.9 (2.9) and 4.1 (2.8) respectively, *P*=0.002).  Table 2 shows that teaching staff had significantly twice or more the odds of being satisfied with the curriculum.

Students had significantly higher scores than teaching staff when they reflected on the curriculum as being in a form “that is required by the university,” “that avoids the risks associated with changing to other types of curricula,” “because there were no other alternatives,” and because there was “lack of awareness of other curricular models” (mean = 7.2, 6.8, 6.2 and 5.5 compared to 5.7, 4.9, 4.8, and 3.6) (Table 3).

## 4. Discussion

At COD-IAU, teaching staff expressed higher satisfaction levels with the curriculum than students. The highest satisfaction level was associated with the curriculum preparing graduates for professional life, and the least satisfaction level was associated with promoting students' engagement with others. Also, respondents clearly believed that the curriculum was developed because it fits the training of the teaching staff.

Most studies evaluating students and/or faculty satisfaction with their dental education in SA are concerned with either individual specialty/course or comparing a different approach in topic teaching and grading [9].

This study is among few in SA that evaluates the satisfaction levels with the whole dental curriculum. Furthermore, one of the strengths of the study is that it compares satisfaction levels of teaching staff and students, as major stake holders, in the same setting. Senior students (final year) and interns were included since they would be in a better position to provide valid feedback compared to junior students who are not yet exposed to full program components. Although the study was conducted in SA, it is safe to assume that similar factors operate in schools with the same setting in various parts of the world. There is increase in dissatisfaction with traditional curricula as reflected by several studies conducted in SA and investigating the introduction of contemporary teaching and assessment tools [10–13].

Radical changes in curricular contents and teaching methods must be calculated.

The students' levels of satisfaction reported in our study are like those of Cardall et al. who found that “curriculum” represented the highest portion (13%) of all negative comments about the educational experience of undergraduate students in five US dental schools [14]. They also noted that this negative perception of the curriculum significantly reduced the morale score of the students. In our study, continuity of training and sequence conducive to learning were two other aspects where faculty markedly expressed greater satisfaction levels compared to students. This was also reported by Lanning et al. who, despite students' satisfaction with the reformed curriculum, still thought that the sequence of courses and the pace at which they progressed from their preclinical courses to clinical experience were hectic [15]. Similarly, students at a variety of different schools in North America expressed concerns about instructional quality in some areas of their curriculum and that teaching and assessment focused on memorization [16]. In another study in India, students expressed the need to improve competence of graduates, introduce general dentistry practice training, and enhance their confidence to treat child patients [17].

Responding students in this study expressed low satisfaction with the way the curriculum developed critical and analytical thinking. On the other hand, the teaching staff were 40% more likely to be satisfied with the curriculum because it develops the ability to think critically and to solve problems. Unfortunately, it is not feasible to objectively assess the controversy but for the sake of improvement, COD may consider lessons learned, for example, from the Critically Appraised Topics (CATs) initiative, to enhance students' capacity in critical appraisal that can be applied during their practice career [18].

In this study, the significantly higher satisfaction ratings of teaching staff may be explained by their feeling of ownership of the program and subsequently the obligation to defend it. They overestimated their students' abilities indirectly, by indicating satisfaction with areas in the curriculum such as evidence-based learning that is scarcely employed in taught courses. In fact, the curriculum allocates ≤2% of total contact hours to these areas, which suggests that the satisfaction of teachers does not align with this part of the curriculum design.

### 4.1. Limitations of the Study

The views of senior graduates, postgraduate programs directors, and employers could add more depth to our understanding of the effectiveness of the curriculum. Another important perspective is the extent to which satisfaction aligns with and supports adherence to international benchmarks.

Faculty members must be encouraged to provide an objective input that can guide the development of the curriculum they will, eventually, deliver.

## 5. Conclusions

In our study, teaching staff and students' satisfaction levels with the curriculum were significantly associated with their perception that the curriculum produces competent graduates. As the standards for competence change, there will be a need to modify curricula to develop these competences. The areas with low students' satisfaction levels were associated with promotion of engagement with others and development of critical thinking, EBD, and problem-solving skills. These areas should be the focus of future improvement actions so that the curriculum is better designed to prepare competitive graduates with competences aligned with the requirements of the international benchmarks.

## Figures and Tables

**Table 1:** Regression analysis for the relation between reasons the existing curriculum is implemented and level of satisfaction.

	Regression coefficient (95% CI)	
	Univariate regression	Multivariate regression
Teacher vs student	1.86 (0.70, 3.02)^*∗*^	1.74 (0.32, 3.17)^*∗*^
Do-able given existing training of teaching staff	0.35 (0.04, 0.65)^*∗*^	0.09 (−0.25, 0.43)
Successful and prepares competent graduates	0.71 (0.40, 1.02)^*∗*^	0.69 (0.17, 1.21)^*∗*^
Fulfills mission and vision of college	0.40 (0.14, 0.65)^*∗*^	−0.38 (−0.77, 0.02)
Reflects college tradition and has been around for some time	0.50 (0.21, 0.79)^*∗*^	0.11 (−0.28, 0.49)
Follows evidence- based best practices in dental education	0.44 (0.19, 0.69)^*∗*^	0.24 (−0.12, 0.60)
Represents the final product of negotiations between departments	0.32 (0.05, 0.59)^*∗*^	0.01 (−0.33, 0.35)
No other alternative was proposed	−0.11 (−0.32, 0.09)	0.04 (−0.26, 0.34)
Not aware of any other model of curriculum exists	−0.15 (−0.36, 0.06)	−0.15 (−0.46, 0.16)
Risks associated with changing into any other alternative	−0.06 (−0.25, 0.14)	0.03 (−0.19, 0.25)
Required by university regulations	0.04 (−0.19, 0.27)	0.01 (−0.25, 0.27)

Adjusted R2 for multivariate model = 0.23. ^*∗*^Statistically significant at *P* ≤ 0.05. CI: confidence interval.

**Table 2:** Satisfaction with various aspects of the existing curriculum.

	Teachers	Students	OR
	%	%	
Promote engagement with others	30	14	2.67
Develops analytic skills	33	31	1.09
Develops EBD skills	34	26	1.51
Develops critical thinking^*∗*^	42	22	2.60
Develops self-learning	44	37	1.31
Develops problem-solving	46	29	2.03
Helps manage community needs	48	35	1.69
Presence of integrated courses^*∗*^	59	38	2.32
Allows continuity of training^*∗*^	60	30	3.56
Develops communication skills^*∗*^	60	35	2.80
Sequence helps student learning	70	52	2.18
Prepares for professional life	71	59	1.70

OR: odds ratio. ^*∗*^Statistically significant at *P* ≤ 0.05.

**Table 3:** Importance of various factors affecting why existing curriculum is being implemented according to the two groups.

	Teachers	Students
	Mean	Mean
Fulfills college mission and vision^*∗*^	8.1	6.3
Reflects colleges tradition^*∗*^	8.0	6.6
Prepares competent graduates	7.7	7.1
Doable given teaching staff training	7.3	7.0
Follows best practices in dental education	6.5	6.6
Negotiation product between departments	6.4	6.3
Required by university regulations^*∗*^	5.7	7.2
High risk associated with changing to other alternative^*∗*^	4.9	6.8
No other alternative proposed^*∗*^	4.8	6.2
Not aware other curricular models exist^*∗*^	3.6	5.5

^*∗*^Statistically significant difference at *P* ≤ 0.05.

## Data Availability

Data are available from the authors upon reasonable request.
